# A Novel Approach to Potentially Improving Soft-Tissue Sarcoma Survival: Prophylactic Lung Radiotherapy Inhibits Growth of Lung Metastases and Prolongs Survival in a Murine Soft-Tissue Sarcoma Model

**DOI:** 10.7759/cureus.76334

**Published:** 2024-12-24

**Authors:** Alexander Ruditsky, Kalin Fisher, Kayla Tighe, Jasmine B'lanton, Xinrong Ma, Kai Jiang, Kevin Byrne, Brandon Carter-Cooper, Andrea Casildo, Antonino Passaniti, France Carrier, Rena Lapidus, Katharina Richard, Michael E Kallen, Vincent Y Ng

**Affiliations:** 1 Department of Orthopaedics, University of Maryland, Baltimore, USA; 2 Translational Laboratory, University of Maryland Greenebaum Comprehensive Cancer Center, Baltimore, USA; 3 Cancer, National Cancer Institutes, Frederick, USA; 4 Department of Radiation Oncology, University of Maryland, Baltimore, USA; 5 Translational Radiation Sciences, University of Maryland Greenebaum Comprehensive Cancer Center, Baltimore, USA; 6 Department of Pathology, University of Maryland, Baltimore, USA

**Keywords:** lung metastasis, murine model, prophylactic, radiation, soft-tissue sarcoma

## Abstract

Background: Circulating tumor cells and clusters (CTC) from soft-tissue sarcoma (STS) that become entrapped in the lung can form micro-metastases and lead to pulmonary metastatic disease. Many patients with localized high-risk STS later develop metastases. Radiation is effective at reducing local recurrence by eradicating microscopic infiltration and satellites in the reactive zone surrounding the primary tumor. Prophylactic lung irradiation for patients with high-risk STS is a novel concept to potentially reduce the appearance of macroscopic metastases and improve survival. A proof-of-principle study was performed based on a novel approach: prophylactic lung radiation after resection of the primary tumor to address microscopic pulmonary deposits from CTC.

Methods: Immunocompromised mice and luciferase-expressing human fibrosarcoma (HT-1080-Luc) cell lines were used. In phase 1, HT-1080-Luc cells were injected into the tail vein to simulate CTC for the development of pulmonary metastases. Whole-lung irradiation (WLI) was then performed in the treated mice prior to the appearance of macroscopic metastases. In phase 2, a flank tumor was established to simulate a primary STS, followed by a tail-vein injection of HT-1080-Luc cells. Treatment groups included surgical removal of the primary STS and hemithoracic irradiation (HTI). Body weight and bioluminescence data were obtained and the mice were euthanized on Day 31 (phase 1) and Day 15 (phase 2) or when they reached 20% weight loss.

Results: In phase 1, prophylactic WLI increased survival and decreased pulmonary metastases. In phase 2, prophylactic HTI (left lung) decreased pulmonary metastases compared to controls. Lung histology showed dramatically decreased growth and number of established metastases with HTI. Resection of the primary tumor did not affect the growth of metastases.

Conclusion: Prophylactic WLI after resection of the primary tumor to inhibit the growth of pulmonary metastases from previously entrapped CTCs may be a promising approach to improve survival for patients with localized high-risk STS.

## Introduction

Approximately 12,000 patients are diagnosed with soft-tissue sarcoma (STS) annually in the USA [1]. Based on the presentation and natural history of the disease, the most important clinical dilemma in STS is the development of pulmonary metastases during the first few years after successful treatment of the primary tumor. High-grade STS first presents as a localized primary tumor in >90% of cases without clinically evident distant metastasis, but approximately 50% of patients with a large, high-grade STS eventually develop metastatic disease at a median of 10-12 months [2,3]. Radiation to the primary tumor has been shown to decrease the rate of local recurrence after surgical resection, presumably by eradicating the infiltrative margins of the tumor and any nascent satellite lesions present in the surrounding reactive zone [4]. Systemic regimens such as anthracycline-based chemotherapy, molecular-targeted therapy, or immunotherapy have limited efficacy in preventing distant relapse [5]. Localized STS is considered curable, but once it manifests as radiographically detectable metastatic disease, it is typically incurable except in rare oligometastatic circumstances [6,7]. Efforts over the past 50 years to prevent metastases have been largely unsuccessful.

Since pulmonary metastases represent the leading cause of mortality in patients with STSs, this study sought to explore an innovative strategy to mitigate this challenge. It has been shown that STS, like many other solid malignancies, shed numerous circulating tumor cells and clusters (CTC) [8-10]. These CTCs become entrapped in the lungs and, if they are able to colonize successfully, eventually become micro- and macro-metastases. Resection of the primary tumor curtails the release of new CTCs, but later-appearing metastases are likely the result of previously disseminated CTCs and nascent pulmonary colonies. The senior author (VYN) hypothesized that there may be a window of opportunity to reduce the risk of distant relapse in the period shortly after resection of the primary tumor. Similar to the paradigms of administering chemotherapy to patients with bone sarcoma to reduce future metastases and of providing neo/adjuvant radiation to the primary tumor in STS to inhibit local recurrence, prophylactic whole-lung irradiation (WLI) to eradicate any nascent pulmonary colonies borne of previously disseminated and subsequently entrapped CTCs may reduce the number of patients with high-risk STS who develop incurable lung metastases. Because the lungs are the predominant site of metastatic involvement for STS and microscopic colonies may be more sensitive to relatively low doses of radiation, there could be a rationale to consider this novel treatment strategy for patients with localized STS identified as high-risk for distant relapse after resection. The purpose of this study was to provide proof-of-concept in a mouse model. The primary objective of this study was to evaluate the efficacy of prophylactic lung radiotherapy in reducing pulmonary metastases and improving survival in a murine model of high-risk STS. In phase 1, the objective was to demonstrate that prophylactic lung radiotherapy can reduce the growth of pulmonary metastases in mice injected with human tumor cells. In phase 2, the objective was to evaluate the impact of prophylactic lung radiotherapy following primary tumor resection on inhibiting pulmonary metastases.

## Materials and methods

The experiment was conducted in two phases. In phase one, a murine model with pulmonary metastasis of STS was developed and WLI was administered. The endpoints were the development of pulmonary metastases as measured by bioluminescence and reaching the humane endpoint or survival of the mouse. In phase two, a primary STS flank tumor with simulated CTC was developed and surgical resection of the flank tumor and/or prophylactic hemithoracic irradiation (HTI) was performed. The endpoint was the development of pulmonary metastases as measured by bioluminescence and histologic examination. All activities were reviewed and approved by the Institutional Animal Care and Use Committee (IACUC) under protocol #1021001.

Randomization of mice bearing primary tumors was stratified by tumor volume (preceding treatment). Tumor volumes were calculated, for group randomization and on each subsequent measurement day, using the ellipsoid formula with external diameter measurements obtained using electronic calipers, rounded to the nearest 0.1 mm. For each independent experiment, one technician performed all caliper measurements to reduce variability stemming from the measuring technique. Mice withheld from subcutaneous tumor cell inoculation to serve as controls were randomized by body weight.

Mice were housed at an Association for Assessment and Accreditation of Laboratory Animal Care International (AAALAC) accredited animal facility with veterinary support available at all times. All veterinary guidelines for animal care, radiation, and surgical procedures, including the use of pre-emptive and post-surgical analgesics (subcutaneous 4-5 mg/kg carprofen) and use of anesthetics (inhaled isoflurane: lidocaine chamber (>3-4.5% in oxygen), maintenance via face mask (>1-3% in oxygen), using a prevision vaporizer with scavenger system).

In-vitro cell culture

HT-1080 human fibrosarcoma cells (ATCC #CCL-121, Imanis Life Sciences, Rochester, MN, United States) were transduced with a yellow fluorescent protein (YFP)-luciferase to stably express firefly luciferase (HT-1080-Luc). HT-1080-Luc cells were cultured in Eagle’s Minimum Essential Medium (EMEM), supplemented with 10% FBS. For injection into mice, the cells were trypsinized, resuspended in a complete medium to deactivate trypsin, and washed twice with PBS. After performing a cell count with trypan blue, the cells were resuspended to a final injection volume appropriate for the injection route (5 × 10^6^ cells in 0.1-0.2 mL PBS with 33% Matrigel for subcutaneous (SC) injection, or 0.5 - 1 × 10^6^ cells in 0.2 mL PBS for intravenous (IV) injection).

Phase 1

Sixteen immunodeficient female NSGTM mice (NOD.Cg-Prkdcscid Il2rgtm1Wjl/Sz, The Jackson Laboratory, Bar Harbor, ME, United States), 6-8 weeks old, were acquired and randomly divided into two groups of eight mice. All mice received a lateral tail injection of 0.5 × 10^6^ HT-1080-Luc cells. Group 1 received no radiation while Group 2 received WLI on Days 3, 4, and 5 (6 Gy total, 2 Gy per day) using the Xstrahl (Atlanta, GA, United States) Small Animal Radiation Research Platform (SARRP). The SARRP was utilized using 220 kVp, 0.15 mm Cu additional filtration (0.65 mm Cu half-value layer), and 12 mA at a dose rate of ~2 Gy/min. The mice were anesthetized with >3.0-4.5% isoflurane through an isoflurane vaporizer during radiation and a cone-beam CT was acquired. The treatment planning system, MuriPlan (Xstrahl), was used to define the radiation treatment field and calculate the irradiation time. A motorized variable collimator (MVC) (Xstrahl) was used to set the fields to encompass the lungs while sparing the guts and other organs. Parallel-opposed anterior-posterior and posterior-anterior beams were used to ensure dose uniformity throughout the irradiated lung. The 2 Gy fraction was delivered at a dose rate of 2 Gy/min on each of the 3 days of radiation. Mice were weighed at least three times per week and bioluminescence imaging of living mice was performed under anesthesia using the Xenogen IVIS Spectrum Optical In vivo Imaging System (Caliper Life Sciences, Hopkinton, MA, United States) twice per week, beginning on Day 10 after injection, using the software Living Image 4.7.4 (PerkinElmer, Waltham, MA, United States). The bioluminescent imaging exposure times were 60 seconds for live animal lung detection, 0.5 seconds for animal tumor detection, and 5 seconds for animal lung organ detection. The mice were euthanized on Day 31 or when they reached 20% loss of body weight as a humane endpoint. Lungs were removed from the carcasses after euthanasia and bioluminescent imaging was obtained ex vivo.

The rationale for using the dose regimen of 2 Gy for 3 days for the whole lung was that in mice, a total of 10 Gy in five fractions was required to initiate the development of lung fibrosis following WLI [11]. To avoid this complication, the regimen of three fractions of 2 Gy each for a biological effective dose of 8.68 Gy assuming an α/β ratio of 4.48 Gy for normal mouse lung was used.

Phase 2

Forty immunodeficient male NRGTM mice (NOD.Cg-Rag1tm1Mom Il2rgtm1Wjl/SzJ, The Jackson Laboratory, Bar Harbor, ME, United States), 8-10 weeks old, were acquired. The treatment plan is described in Table 1. Eight mice were reserved for the control group (Group 1), and the remaining 32 mice were inoculated subcutaneously (SC) on the right flank with 5 × 10^6^ HT-1080-Luc cells in 0.1 mL PBS containing 33% Matrigel. Previous preliminary work by this group (unpublished) confirmed existing literature [12] that the presence of such subcutaneous flank tumors does not produce spontaneous pulmonary metastases. Tumor size was tracked using electronic calipers. Six days after SC cell injection, the majority of primary tumors reached a size of 95-242 mm^3^ (average 141 mm^3^), and mice with tumors that engrafted at a different rate or already showed signs of tumor necrosis were removed from the study (euthanized). All mice, including Group 1 without primary tumor and 26 mice with primary tumors in the selected size range, were injected IV in the tail vein with 1 × 10^6^ HT-1080-Luc cells in 0.2 mL PBS. Three days following IV cell injection, the pool of 26 mice was randomly assigned to treatment Groups 2-5, based on the size of their primary tumor (to equalize the tumor burden prior to treatment; average tumor volume 200 mm^3^), and mice in Groups 3 and 5 had their flank tumors surgically resected. Six days following IV cell injection, 8 of 18 mice that did not meet humane endpoints (Groups 4 and 5) underwent HTI to the left lung with photons for 15 Gy × 1 fraction on the SARRP following similar procedures described in phase 1. However, HTI instead of WLI was used in phase 2 of the experiment to help improve radiation tolerance, increase the pulmonary dose, and provide an internal (contralateral lung) control. The MVC was used to set the fields to encompass the entire left lung while sparing the contralateral lung, guts, and other organs. Mice were then imaged with bioluminescent imaging twice per week. The mice were weighed daily and the primary tumors measured three times per week. Mice were euthanized at 15 days after IV injection. Lungs were removed from the carcasses after euthanasia and bioluminescent imaging was obtained ex vivo. Samples were prepared for further histological analysis.

**Table 1:** Treatment Groups for Phase 2 IV: intravenous injection of tumor cells into tail vein; SC: subcutaneous tumor via injection in the flank with tumor cells; HTI: hemithoracic irradiation to left lung

Group	N planned	Treatment	N Final
1	8	IV injection (Control)	8
2	8	SC tumor + IV injection	5
3	8	SC tumor + IV injection + tumor resection	5
4	8	SC tumor + IV injection + HTI	4
5	8	SC tumor + IV injection + tumor resection + HTI	4

The rationale for the radiation dose was that a single 15 Gy dose to the whole thorax increases the risk of developing fibrosis in mice [11]. In humans, WLI of 12 to 20 Gy with daily dose fractions between 1.5-2.0 Gy were well tolerated [13]. By irradiating a single lung in a mouse, the efficacy of a higher dose to prevent lung metastasis could be measured and the non-irradiated lung could be used as an internal control.

Data analysis

Data were expressed as mean ± standard error and analyzed with GraphPad Prism Version 7 (GraphPad Software Inc., San Diego, CA, United States). Statistical significance between groups was determined using an unpaired t-test. A value of p <0.05 was considered statistically significant. Based on prior studies, six mice were estimated to be sufficient to detect a 20% difference between groups with a power of 80% and alpha 0.05. Analysis of variance testing was utilized to determine differences between groups in phase two.

## Results

Phase 1

**Figure 1:**
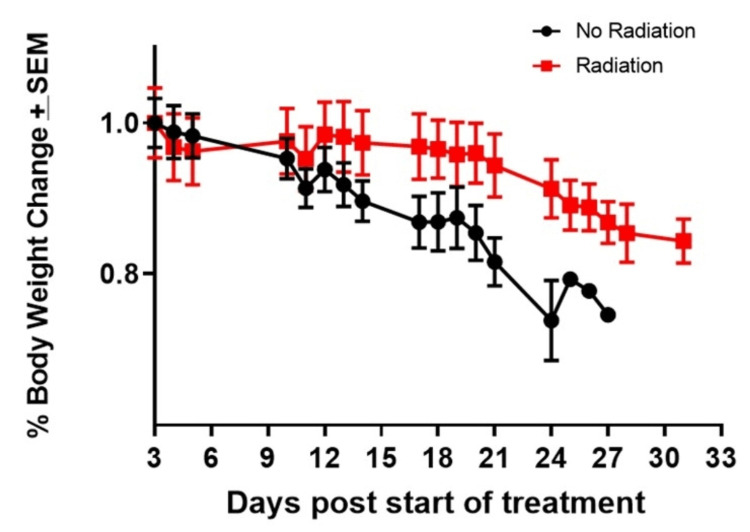
Body weight percent change in radiation and control groups The figure was created by the authors of this article.

Mice were randomized by body weight just prior to receiving the sham (Group 1, N = 8, mean body weight = 25.46 g, SD 2.37 g) or radiation treatment (Group 2, N = 8, mean body weight = 25.49 g, SD 3.34). Initially, the radiation treatment group showed a slightly faster decline in body weight after three consecutive days of radiation (indicated by arrows in the graph, Figure 1) compared to the control group, however, this was not statistically significant (mean rad -0.96 g +/- 0.15 (4%) vs mean sham -0.44 g +/- 0.15 (2%)), (P=0.72). After Day 5, the control group lost weight more consistently and more rapidly throughout the duration of the experiment. Weight loss differences between the two groups strongly trended toward statistical significance until Day 17, at which time mice in the control group began dropping below the 20% weight loss threshold and were euthanized (mean difference between groups at Day 17, -10.13% (-2.55 g); 95% confidence interval (CI) -14.50 to -5.75 % (-3.67 to -1.43 g; P=0.0003).

**Figure 2:**
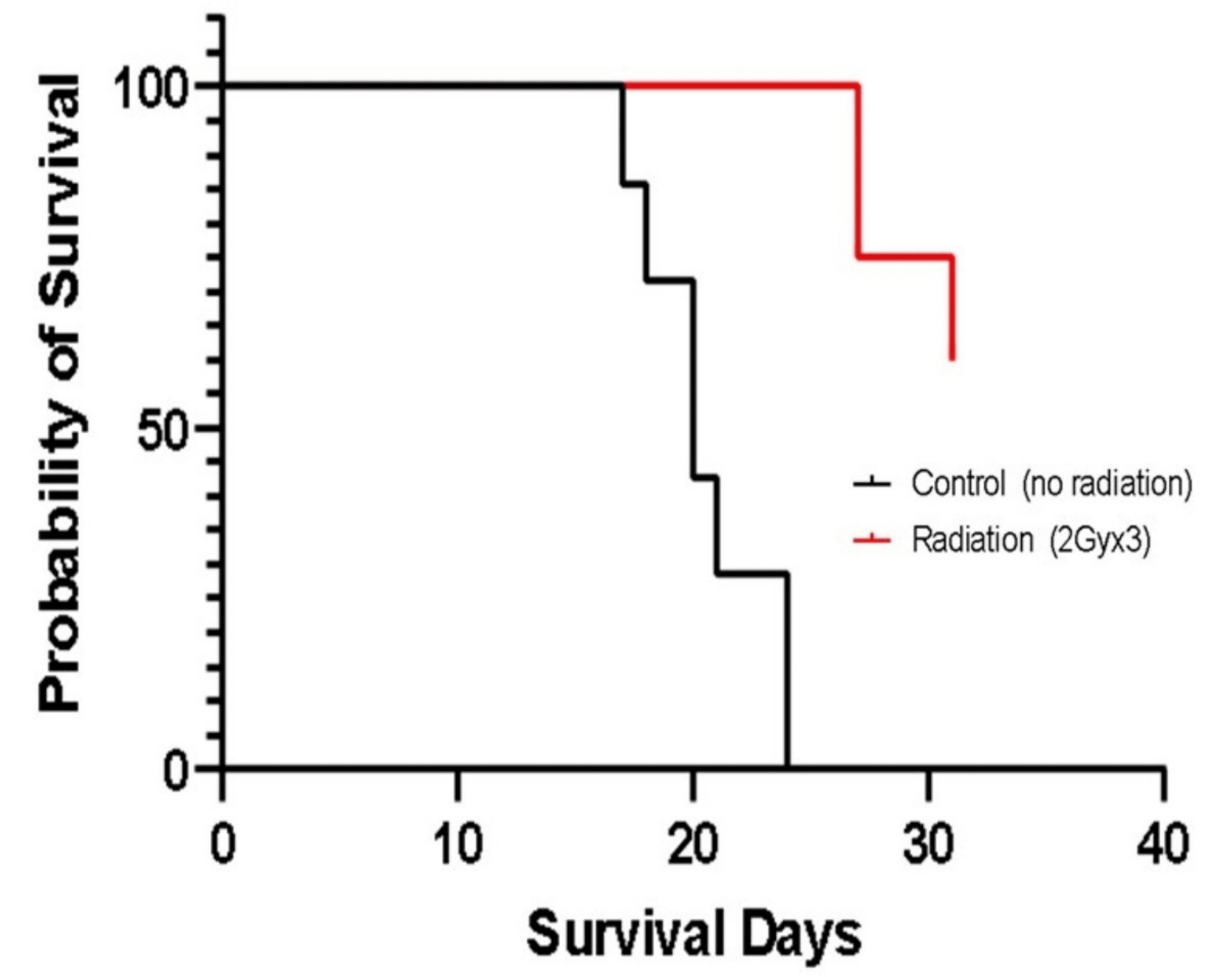
Survival in treatment and control groups The figure was created by the authors of this article.

All mice in the control group were euthanized due to meeting humane endpoint criteria between Day 17 and Day 27. Conversely, in the radiation treatment group, the first mice requiring euthanasia due to reaching humane endpoints started on Day 27. A total of three mice in the radiation group were euthanized, and the remaining five survived until the 31-day time point (Figure 2), with a delay to euthanasia estimated at 9 days for radiation treatment vs. sham.

**Figure 3:**
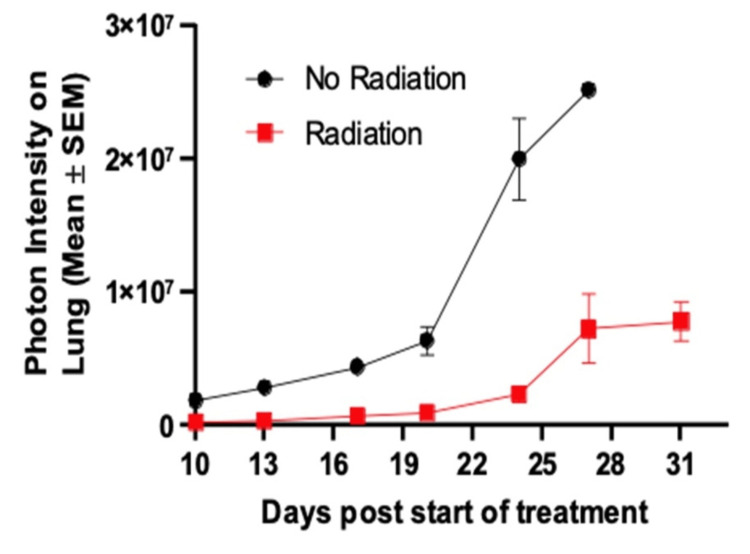
Photon intensity of lungs vs. time The figure was created by the authors of this article.

**Figure 4:**
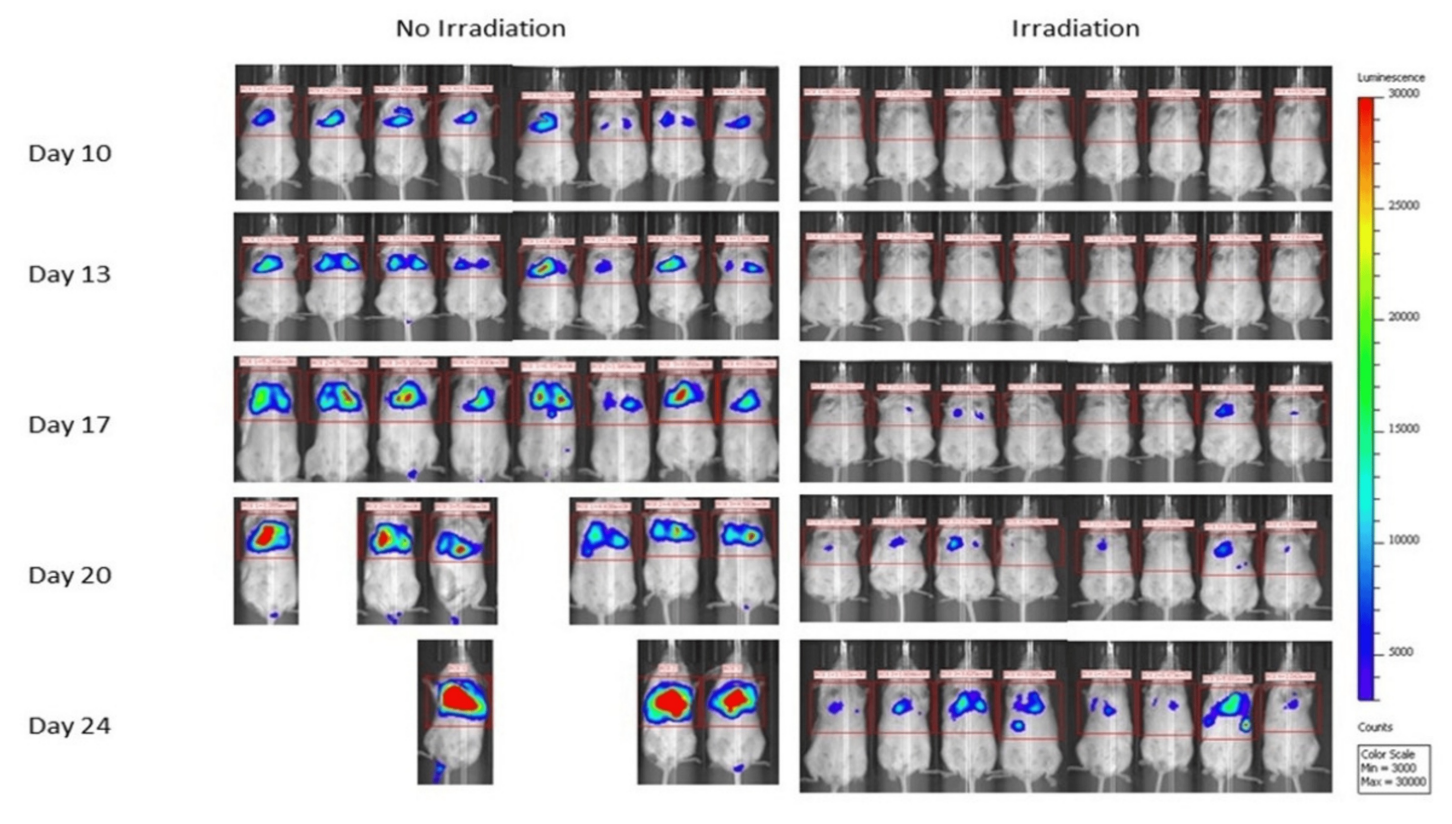
In-vivo bioluminescent imaging over time The figure was created by the authors of this article.

Bioluminescent imaging demonstrated increased tumor burden in the control group compared to the radiation treatment group at all time points, beginning on post-injection Day 10 (average 1.83 × 10^6^ vs 2.28 × 10^5^ photons) (Figure 3). On Day 10, the mean difference in photon intensity between groups was -1.60 × 10^6^ (95% CI, -1.18 × 10^6^ to 2.03 × 10^6^; P<0.0001). In addition, this treatment effect became larger as the experiment continued, suggesting a lasting effect of the radiation (mean difference in photon intensity on Day 24 = 1.77 × 10^7^; 95% CI: 1.32 × 10^7^ to 2.22 × 10^7^; P<0.0001). Mice in the radiation treatment group demonstrated the largest increase in photon intensity between Day 24 and 27 (mean increase 4.99 +/- 11.5 × 10^6^), which coincided with the greatest drop in body weight (Figure 4).

Phase 2

**Figure 5:**
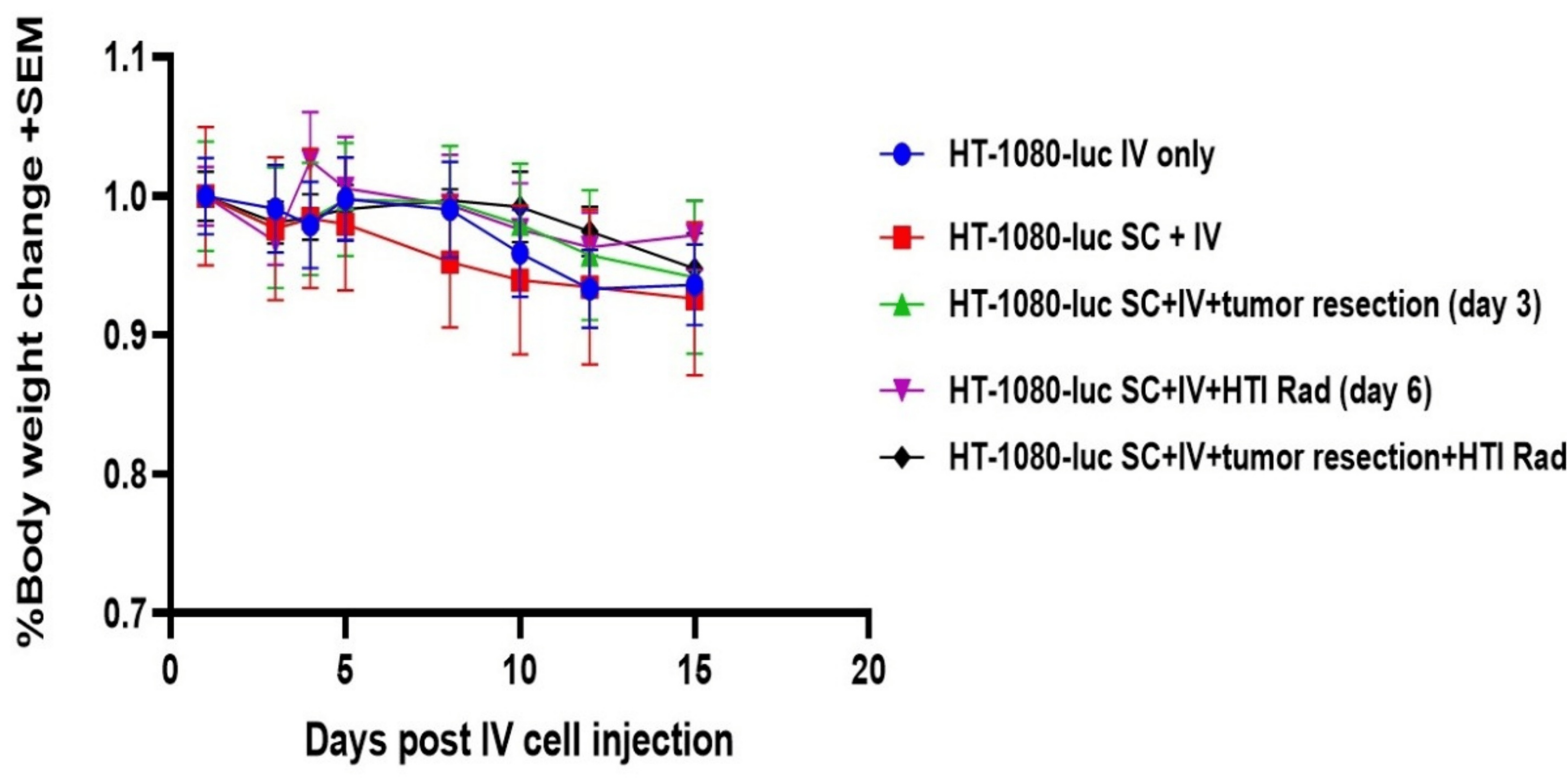
Percent body weight change The figure was created by the authors of this article.

**Figure 6:**
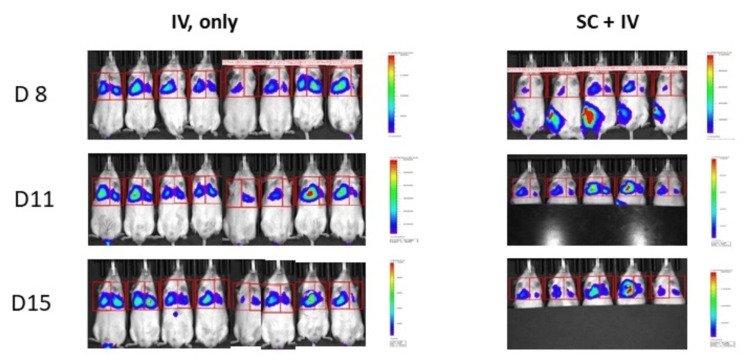
Bioluminescent imaging demonstrating the development of pulmonary metastasis in all control mice that received IV injection or subcutaneous tumor plus IV injection and did not receive radiation

**Figure 7:**
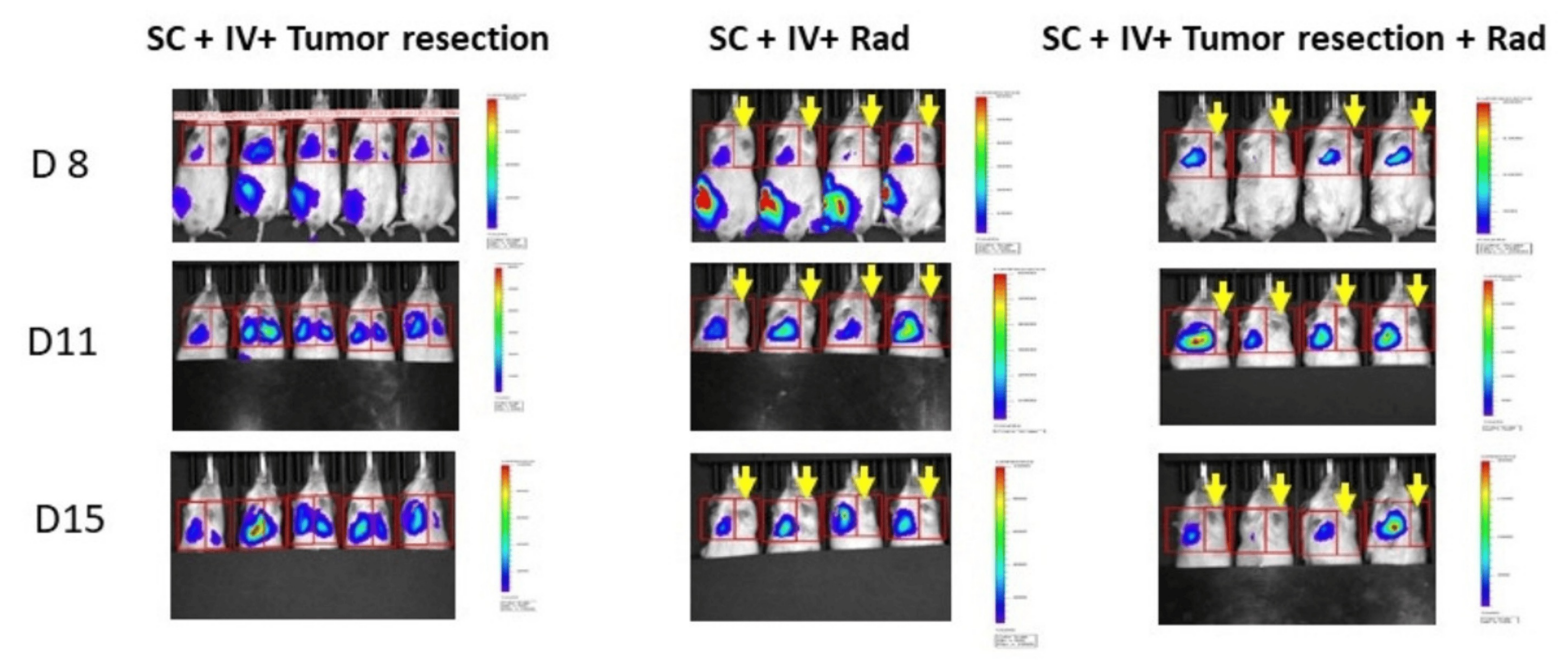
Bioluminescent imaging demonstrating minimal to no signal in the left lungs that received radiation Yellow arrows indicate the lungs that received radiation.

**Figure 8:**
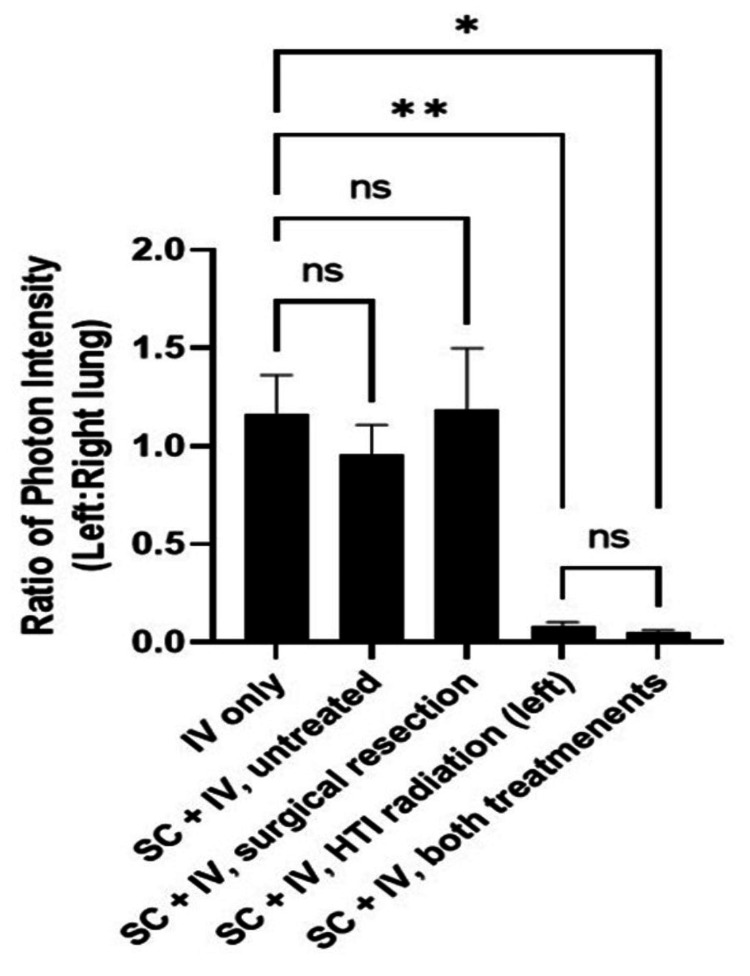
Ratios of pulmonary metastases between left and right lung measured by photon intensity at euthanasia ns: not significant *p<0.02, **p<0.01 The figure was created by the authors of this article.

There was no statistically significant difference in body weight change between treatment groups throughout the experiment suggesting that the treatments including surgery and HTI were well tolerated (Figure 5). Pulmonary metastasis developed from the CTC simulated by IV tail injection in all groups of our model (Figure 6). Photon intensity was observed to be higher in the non-irradiated right lung for all mice receiving HTI (Figures 6-7; primary tumors were covered with an opaque sheet to prevent saturation of the long-exposure images to detect lung metastases). The left lung demonstrated significantly lower photon intensity in treatment Groups 4 and 5 (Figure 7). There was no significant difference in pulmonary photo-intensity in groups that underwent tumor resection versus those that did not (Groups 2 and 3). After euthanasia, the bioluminescence of the right and left lungs was measured ex vivo and there was a significant difference seen between the mice that received HTI (Groups 4 and 5) vs those that did not (Figure 8; One-Way ANOVA p=0.0019, Sidak’s post-hoc comparison “IV only” vs. “SC + IV”, p = 0.9598; “IV only” vs. “SC + IV + tumor resection”, p > 0.9999; “IV only” vs. “SC + IV + HTI Rad”, p = 0.0066; “IV only” vs. “SC + IV + both treatments”, p = 0.0123).

**Figure 9:**
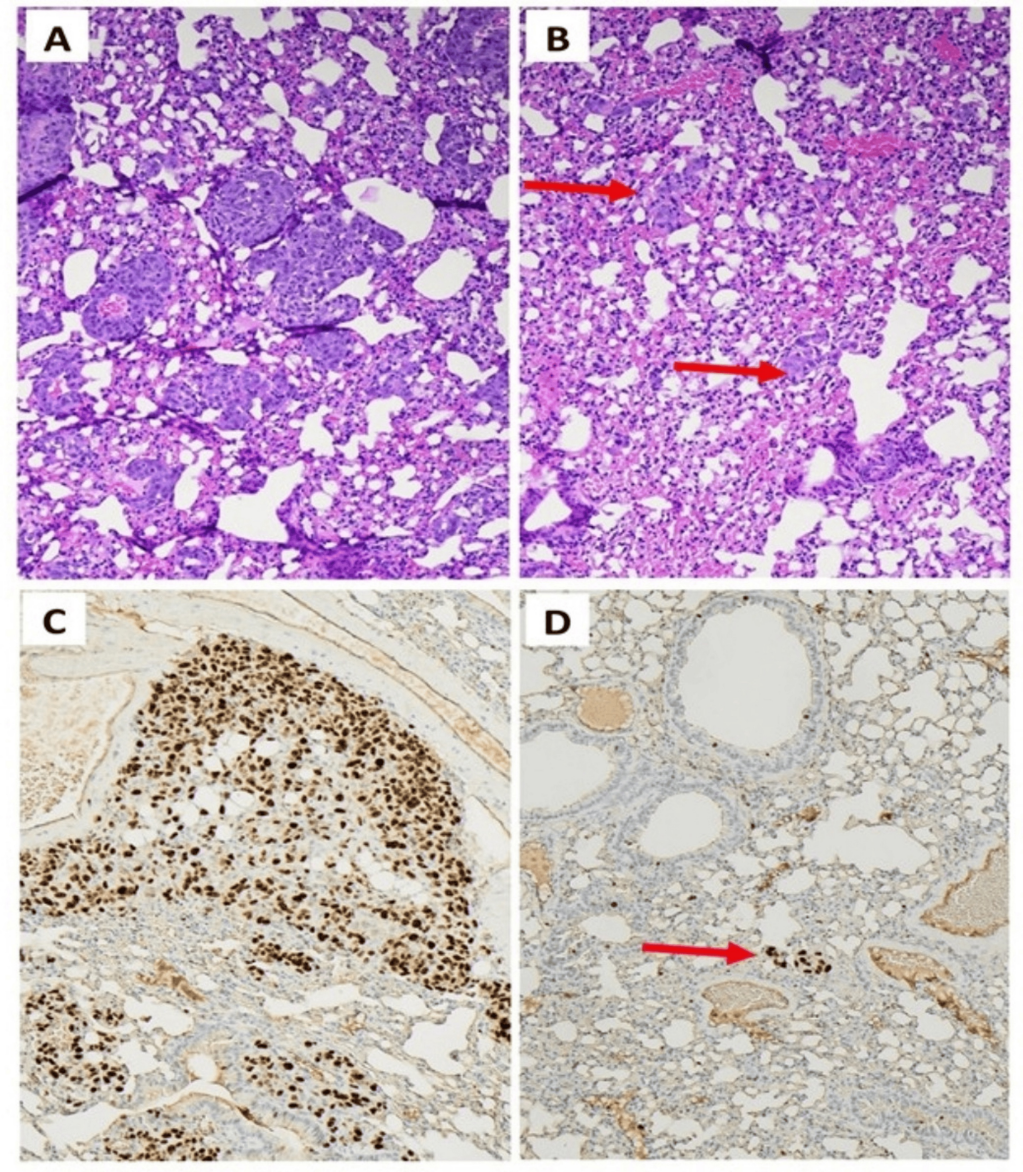
Histologic images at 100× magnification of the right and left lungs of a mouse that had an establishment of a flank tumor and IV injection of HT-1080-Luc followed by resection of the flank tumor and HTI to the left lung (A) Right lung demonstrates innumerable tumor nests, arranged around blood vessels, and of varying sizes from very small to large aggregates, diffusely throughout the lung parenchyma at 100× magnification. (B) The left lung demonstrates relatively few minute scattered tumor nests (red arrows) scattered throughout the lung parenchyma at 100× magnification. In contrast to the right lung, the left lung demonstrates a marked decrease in tumor nest frequency and size, and a lack of angiocentricity. (C) Ki67 immunohistochemical stain from the right lung, demonstrating a high proliferative index within tumor cells which form large aggregates and show angiocentricity at 400× magnification. (D) Ki67 immunohistochemical stain from the left lung, which shows a single minute cluster of tumor cells (red arrow) with a high proliferative index, and a marked decrease in tumor burden compared to the right lung at 200× magnification.

Histologic review of the lung specimens after euthanasia showed innumerable metastatic foci of varying size in all mice and lungs that did not receive radiation (Figure 9). The irradiated lungs had sparse foci of tumor cells existing along the airspaces lined with minute capillaries whereas the non-irradiated lungs had large tumor growths obliterating the nearby airspaces and demonstrated significant angiocentricity. Angiocentricity around native vessels is typically characteristic of high-grade and rapidly growing malignancies; it was only seen in the non-irradiated lungs. The tumor burden seen in the non-irradiated mouse lungs would be equivalent to extremely advanced disease in humans and vastly exceeds what would typically be seen clinically in pulmonary resections from living patients. No radiation-related histologic changes were seen in the lung parenchyma, aside from the striking decrease in tumor burden.

## Discussion

Development of pulmonary metastases within the first couple years after definitive treatment of the primary tumor is currently the largest challenge for the successful management of high-grade STS. Metastatic STS has a poor prognosis and long-term survival is rare [14]. The efficacy of chemotherapy, immunotherapy, or molecular-targeted therapy is controversial and there lacks a proven method to significantly decrease the risk of distant relapse after treatment of localized disease [15]. This study offers proof-of-principle in a mouse model with human STS for a highly novel approach to addressing this issue. Prophylactic pulmonary radiation was shown in mice to improve survival and inhibit metastatic colonization from CTC and subsequent outgrowth of metastases from high-grade STS.

Although the radiated lungs still demonstrated the presence of some CTC colonization and eventual growth of metastatic colonies in this mouse model, it should not be interpreted that the potential clinical effect would be limited to delaying rather than preventing metastatic growth. The HT-1080-Luc STS is a highly aggressive fibrosarcoma and the amount of tumor burden injected via the tail vein creates likely a greater amount of CTC and clusters than that seen in clinical cases of primary high-grade STS releasing spontaneous CTC. The pulmonary radiation was able to reduce the successful colonization of CTC significantly and in lower volumes of CTC, may prevent it altogether.

There are some limitations to this murine proof-of-principle study. First, there are inherent differences between murine models and human biology in terms of the behavior of the primary tumor, CTC, and metastasis and the effect of radiation on lung tissue. Second, although the clinical course of sarcoma progression in the murine model is more rapid than in humans, this study had a relatively limited follow-up period to examine the long-term effects of radiation on cancer-related survival and on lung function. Third, because STS represents a heterogeneous group of tumor types, the effect of prophylactic pulmonary radiation may vary depending on subtype and patient-related factors. Because of these limitations, further murine model experimentation and eventual human clinical trials would be needed in order to validate these findings and evaluate safety.

Patients with high-grade STS typically receive radiation and surgical resection of the primary tumor, but aside from removing the source of CTC, local control may not influence the risk of distant relapse [16]. The current paradigm for the treatment of high-grade STS is largely based upon two traditional tenets. First, patients can be distinguished into binary categories: those with localized disease and those with metastatic disease. Second, patients who present with ostensibly localized disease often develop metastases after otherwise successful treatment of the primary tumor. The most widely agreed upon predictive factor for which patients with high-grade STS will ultimately cross the Rubicon from curable localized to incurable metastatic disease is tumor size [17-19]. However, it should be noted that although the risk of developing metastatic disease increases with each increment of tumor size, it reaches neither 0% risk nor 100% risk on either end of the spectrum for very small or very large tumors, respectively.

The hypothesis of this study that prophylactic pulmonary radiation for those patients with ostensibly localized disease can reduce the risk of developing radiographically visible and clinically incurable metastases is based upon a slightly different paradigm for STS. Solid malignant tumors, including STS, have been shown to release millions of CTC and clusters into the bloodstream [8-10]. These CTC and clusters are mechanically trapped in the pulmonary vasculature [20,21]. Relatively few CTC clusters travel beyond the lungs into other organs such as the liver or bone, evinced by the pulmonary predominance of metastatic locale [22]. The vast majority of CTCs perish within 24-48 hours and only a very small number remain viable and even fewer are able to colonize and grow at their site of dissemination [22-26]. Circulating clusters of tumor cells have a greater capacity for proliferation, survival in the lung, and formation of metastases but are released with much lower frequency than individual CTC and spend a shorter time in circulation [27-31].

The timing of the appearance of STS metastases follows a distinct pattern. The majority of patients present with no definitive metastases and distant relapse are radiographically diagnosed within 2 years after treatment of the primary tumor. Interestingly, the duration of symptoms or delay in diagnosis of STS prior to presentation has not been shown to be a consistent and reliable predictor of metastases [32-36]. This counterintuitive observation, combined with the fact that size is a well-recognized risk factor for metastases, have historically prompted theories that the presence of the primary tumor suppresses the appearance of metastases and that its removal triggers the outgrowth of lung nodules. The mechanisms to explain this have included concomitant immunity, angiogenic factors, and surgery-related inflammation [37-39].

An alternative explanation may be more plausible. Many high-grade STS patients present with a mass that they may or may not have noted to be present for a variable amount of time as relatively small or innocuous, but then recently grew rapidly and came to their attention over the past 4-8 months. This growth was alarming to them and prompted the majority of them to seek specialized care within this approximate timeframe [18]. If the patient ignored the symptoms long enough, the primary tumor often plateaued in size once it reached its biological or anatomic constraints. Occasionally, a patient may present with a relatively small mass prior to any rapid growth. Thus, the typical high-grade STS tumor growth is represented by an S-shaped or Gompertzian growth curve with an initial horizontal asymptotic phase (during which the patient is usually unaware or ignores the relatively small mass), an exponential rapid growth phase (which leads the patient to seek care), and a final horizontal asymptotic phase (if the patient ignores the mass for long enough). For any given tumor, the number of CTC and clusters released is proportional to its size [40]; the linear slope of increasing metastatic risk based on size can be attributed to the fractal nature of solid tumors. Hence, regardless of the duration of symptoms noted by the patient when the tumor remained small in the initial part of the Gompertzian Curve, the key factor is when the diagnosis was made or more importantly, when treatment was initiated relative to the tumor’s exponential growth phase. As the tumor rapidly grows larger during this phase, significantly more CTC are released and the chance of at least some of them colonizing the lungs increases. Once the STS is diagnosed, if the tumor is removed, the source of CTC is eliminated or alternatively, if neoadjuvant radiation to the primary tumor is initiated, the ability of subsequent CTC released to form metastases is significantly reduced [41]. The reason that metastases are usually not visible at the time of presentation but appear within the first 1-2 years after treatment is because any CTCs released during the rapid growth phase of the primary tumor, usually 4-8 months before presentation to the specialist, that have successfully colonized the lungs, require enough time to actually become radiographically detectable. The median tumor volume doubling (TVD) time, i.e., the rate at which the number of cells or volume of tumor doubles, for sarcoma lung metastases is approximately one month [42,43]. It takes about 20 TVD for a single tumor cell to reach 1 mm in diameter and about 10 TVD more to reach 1 cm in diameter [44,45]. Therefore, a CTC cluster released during the rapid growth phase of the primary STS prior to initial diagnosis would likely become radiographically evident around year 1-2 after resection of the primary tumor. If no progressive nodules have been detected by 2 years after removal of the primary tumor, the patient’s prognosis is relatively good, as they either have had no CTC successfully colonize their lungs or the growth rate of any nodules that have yet to appear is very slow [46].

This paradigm reveals a possible opportunity to improve survival for patients who present with localized high-grade STS. Because the majority of pulmonary metastases that would otherwise later appear are likely borne of CTC released within the rapid primary tumor growth phase within 4-8 months before initial diagnosis and are likely still relatively nascent colonies at the time of primary tumor extirpation, irradiation may prevent their future outgrowth. There is significant evidence and rationale that small foci of tumor and subclinical disease are overall less robust and more sensitive to radiation than larger masses [47-50]. Angiogenesis is required for growth beyond 1-2 mm in diameter and relative radioresistance emerges at approximately 200 mm^3^ or 7 mm diameter [44,48]. The efficacy of radiation against microscopic foci of STS is clearly demonstrated by the reduction of local recurrence when radiation to the primary tumor and reactive zone is added to surgery [51,52]. Administering prophylactic WLI to eradicate these incipient CTC colonies prior to their growth into visible metastases may reduce the number of patients who ultimately develop incurable lung metastases. Of note, the timing of prophylactic whole-lung radiation would need to be at least several days after resection of the primary tumor such that any CTC still circulating would have had time to clear out of the bloodstream. Although radiation can kill pulmonary colonies already present, it may theoretically predispose the lungs to colonization of any CTCs becoming newly entrapped after radiation [22,53-56].

There are several potential concerns with prophylactic whole-lung radiation. First, it is possible that patients can relapse at other distant sites. While this may be true and would be unaddressed with this strategy, the natural pulmonary entrapping of CTC provides an opportunity to prevent further spread beyond the lungs in the majority of patients. Second, because not every patient with high-grade STS eventually develops metastasis, this strategy would entail some patients receiving radiation unnecessarily in theoretical retrospect. In order to minimize this, patients would need to be risk stratified using factors that impact not only the likelihood of metastasis such as tumor size, location and rate or chronology of tumor growth relative to diagnosis, histologic features (infiltrative border, necrosis, perivascular invasion), patient smoking status and patient age but also the likelihood to tolerate radiation such as patient comorbidities and concomitant chemotherapy [57,58]. The lungs would need to be carefully evaluated beforehand for the presence of nodules, particularly those greater than 5-6 mm due to presumed radioresistance, with high-resolution CT scans including maximal intensity projection (MIP) sequences [59]. The presence and amount of CTC at the initial diagnosis of STS can be measured to further risk stratify patients [9]. Third, prophylactic lung radiation may have side effects [60]. In Ewing sarcoma, WLI total radiation doses up to 15-18 Gy in 1.5 Gy per fraction have been shown to be relatively well tolerated and to have oncologic benefits in relapsed patients [61,62]. A systematic review found acute lung toxicity in 0-12% of patients including dyspnea and cough (4.5% patients) and severe pneumonitis (1.8%), mainly in patients who received boost radiation, had previous thoracic surgery, were smokers or also were receiving chemotherapy. Pulmonary function testing (PFT) was not always included but several studies reported normal PFT in 40-100% [13]. Modern ultra-fractionated and cardiac-sparing radiation techniques may also minimize toxicity [63]. Sarcoma cells are not felt to be particularly radioresistant compared to carcinoma cell lines and high doses of radiation are not likely necessary for microscopic or pre-metastatic disease [64]. The risk of toxicity with prophylactic radiation should be weighed against the risk and ramifications of metastatic relapse. The minimum dose necessary to induce tumor cell necrosis or simply disrupt the proliferative capacity of the STS deposits would need to be determined [65]. Furthermore, the risks need to be seen in the context that some centers in the USA and many studies utilize neoadjuvant or adjuvant chemotherapy in the setting of localized, high-risk STS despite well-known dose-dependent toxicity and controversial evidence as to its long-term benefit in reducing distant relapse [66]. If prophylactic pulmonary radiation can reduce the likelihood of developing metastasis, the benefits would likely outweigh the risks in high-risk cases. Fourth, prophylactic pulmonary radiation may not prevent the outgrowth of colonized CTC in all patients. However, pursuing a cure should not be an all-or-nothing proposition. Cancer and more specifically, STS are very complex from a genetic, epigenetic, immunologic, behavioral, and evolutionary standpoint for which finding the exact key or set of keys to unlock a total cure may be impossible. However, if prophylactic pulmonary radiation can reduce the risk of metastasis in some patients, it may significantly contribute to what amounts collectively with other risk-reducing interventions to a stochastic sword strike through the Gordian Knot of STS.

## Conclusions

The concept of prophylactic pulmonary radiation in the setting of localized high-risk STS is based upon the paradigm that CTC entrapped in the lungs and nascent microscopic deposits may be eradicated before they can establish colonies and grow into incurable metastases. There may be an effective window of opportunity to administer prophylactic pulmonary radiation shortly after resection of the primary tumor. This hypothetical novel treatment strategy has shown promise in this proof-of-principle murine study. Careful further testing and clinical trials would be needed to determine its safety and clinical efficacy.
